# Effects of VEGF blockade on the dynamics of the inflammatory landscape in glioblastoma-bearing mice

**DOI:** 10.1186/s12974-019-1563-8

**Published:** 2019-10-28

**Authors:** Aurélie Soubéran, Sophie Brustlein, Caroline Gouarné, Lionel Chasson, Aurélie Tchoghandjian, Marie Malissen, Geneviève Rougon

**Affiliations:** 1 CNRS, Institut des Neurosciences de la Timone, UMR 7289, Aix-Marseille Univ, 27 Boulevard Jean Moulin, 13005 Marseille, France; 2 INSERM, CNRS Centre d’Immunologie de Marseille-Luminy, INSERM U1104, CNRS UMR7280, Aix-Marseille Univ, 13288 Marseille Cedex 9, France; 3 CNRS, Institut de Neurophysiopathologie, UMR 7051, Aix-Marseille Univ, 27 Boulevard Jean Moulin, 13005 Marseille, France

**Keywords:** Glioblastoma, Angiogenesis, Innate immune response, Dendritic cell subsets, Spectral in vivo two-photon imaging

## Abstract

**Background:**

Targeting angiogenesis has been and continues to be an attractive therapeutic modality in glioblastoma (GBM) patients**.** However, GBM rapidly becomes refractory to anti-VEGF therapies. Myeloid cell infiltration is an important determinant of tumor progression. Given that VEGF is a modulator of the innate immune response we sought to analyze the dynamics of this response in a mouse model of GBM undergoing anti-VEGF therapy.

**Methods:**

We grafted GL261-DsRed cells in transgenic Thy1-CFP//LysM-EGFP//CD11c-EYFP reporter mice. We combined recurrent spectral two-photon imaging with multiparametric cytometry, immunostaining, and brain clearing to characterize at two critical stages of tumor development (day 21 and day 28 after tumor grafting) the nature and spatial distribution of the innate response in control and bevacizumab-treated mice.

**Results:**

We report that at an early stage (21 day), VEGF blockade has a detectable effect on the number of microglial cells but only a mild effect on the number of infiltrating myeloid cells. At a later stage (day 28), the treatment resulted in a specific adjustment of dendritic cell subsets. In treated mice, the number of monocytes and their monocyte-derived dendritic cells (moDC) progeny was increased by approximately twofold compared to untreated mice. In agreement, by in vivo quantitative imaging, we observed that treatment increased the number of LysM-EGFP cells traveling in tumor blood vessels and doubled the densities of both infiltrated LysM-EGFP monocytes and double-labeled EGFP/EYFP moDC. The treatment also led to an increased density of conventional cDCs2 subset together with a decrease of cDCs1 subset, necessary for the development of anti-tumor immunity. Finally, we describe differential spatial cell distributions and two immune cell-traveling routes into the brain. LysM-EGFP cells distributed as a gradient from the meninges towards the tumor whereas CD11c-EYFP/MHCII^+^ cells were located in the basal area of the tumor. Brain clearing also revealed a flow of CD11c-EYFP cells following the corpus callosum.

**Conclusion:**

We uncovered new features in the dynamics of innate immune cells in GBM-bearing mice and deciphered precisely the key populations, i.e., DC subsets controlling immune responses, that are affected by VEGF blockade. Since despite differences, human pathogenesis presents similarities with our mouse model, the data provide new insights into the effect of bevacizumab at the cellular level.

**Electronic supplementary material:**

The online version of this article (10.1186/s12974-019-1563-8) contains supplementary material, which is available to authorized users.

## Background

Targeting angiogenesis has been and continues to be an attractive therapeutic modality in both newly diagnosed and recurrent glioblastoma (GBM) patients. Vascular endothelial growth factor (VEGF) is the most abundant and important mediator of angiogenesis in GBM [1]. Anti-angiogenesis with Bevacizumab (Bev), an anti-VEGF monoclonal antibody [2], has been used for devascularization to limit the growth of malignant glioma. However, Bev antibody failed to prolong overall survival despite the extension of progression-free survival in three randomized phase III trials [3–5]. These transient benefits are due to elusive mechanisms underlying resistance to the anti-angiogenic therapy [6]. The role of VEGF blocking agents and how to best incorporate them into the treatment paradigm for GBM should evolve if the understanding of their effects and that of subsequent resistance mechanisms improves.

Cancer is closely associated with inflammation. The GBM tumor microenvironment contains innate immune cells in addition to the cancer cells and their surrounding stroma [7]*.* Microglia/macrophages were assumed as one reason for the poor beneficial effect of anti-angiogenic therapy. However, if literature evidences the effects of VEGF on GBM [8], the underlying mechanisms and their impact on microglia/macrophages are not clarified sufficiently and some data are contradictory. VEGF is able to mobilize blood monocytes and microglia cell lines in vitro [9, 10], and microglia/macrophages themselves produce VEGF [11, 12]. Some studies report that anti-angiogenic therapy led to an increase in the amount of microglia/macrophages that conduce to resistance development [13–15]; however, this increase is not documented in terms of kinetics or quantitative data on cell subsets.

In an earlier study [16], we developed an orthotopic GBM model by grafting U87 in nude mice and recapitulating the biophysical constraints normally governing tumor invasion. This model suitable for intravital multiphoton microscopy allowed to repeatedly imaged tumor cells and blood vessels during GBM development in control and Bev treated mice. The treatment massively reduced tumoral microvessel densities but only transiently reduced tumor growth rate [17]. Altogether our results supported the view that GBM growth is not directly related to blood supply but, as proposed by others [18], that tumor angiogenesis and tumor growth could be promoted by inflammation.

In the brain, differential contributions of infiltrating versus resident myeloid populations have been demonstrated in the pathogenesis of GBM. In order to gain insight in the respective involvement of resident microglia and circulating leucocytes across the different stages of tumor development, we devised a clinically relevant syngenic GBM model suitable for intravital dynamic multiphoton imaging by grafting the murine DsRed-GL261 cell line in C57BL/6 multicolor Thy1-CFP//LysM-EGFP//CD11c-EYFP fluorescent reporter mice [19]. In these animals, CFP expression occurs in subpopulations of neurons; EGFP in peripheral myelomonocytic cells including neutrophils, infiltrating monocytes and their progeny; and EYFP in a subset of microglia. They are particularly appropriate for long-term tracking of different types of immune cells in vivo. We showed that invasion of the tumor by microglial CD11c-EYFP^+^ cells dominated early stages of tumor development, then followed by a massive recruitment of circulating LysM-EGFP^+^ cells.

In the present study, we used the above mouse GBM model to assess, by in vivo two-photon imaging combined to immunochemistry and multiparametric cytometry (FACS), how Bev therapy influenced the inflammatory landscape at two critical times of tumor development and to evaluate whether it reprograms the tumor immune microenvironment. Besides uncovering some specific features of the spatio-temporal distribution of recruited subsets of immune cells, our findings strongly support that VEGF blockade has an effect on blood vessels, levels of monocytes traveling in the blood vessels, and the density of myeloid recruited cells. Importantly, Bev modifies the ratios between subsets of DCs and the number of MHCII expressing cells thus possibly the way in which innate response controls the adaptive response.

## Material and methods

### In vivo experiments

#### Animals

To study the immune response induced by the tumor, we had to work in a syngenic model in C57BL/6 transgenic immunocompetent mice. C57BL/6 mice (*n* = 50) were housed in cages with food and water ad libitum in a 12h light/dark cycle at 22 ± 1 °C.

#### Cell culture and gene transfection

GL261 cells, a C57BL/6 murine GBM cell line (National Cancer Institute, Charles River Labs), were transfected with a plasmid encoding for DsRed (pDsRed2-N1, Clontech). Cells that stably express DsRed were selected, cultured, and induced to form spheroids as described [17].

#### Animal model

LysM-EGFP [20], CD11c-EYFP [21], and Thy1-CFP (JAX stock #003710) [22] mice were crossed to obtain triple transgenic mice. The GBM animal model was realized as previously described [16].

#### Experimental timetable

**Fig. 1:**
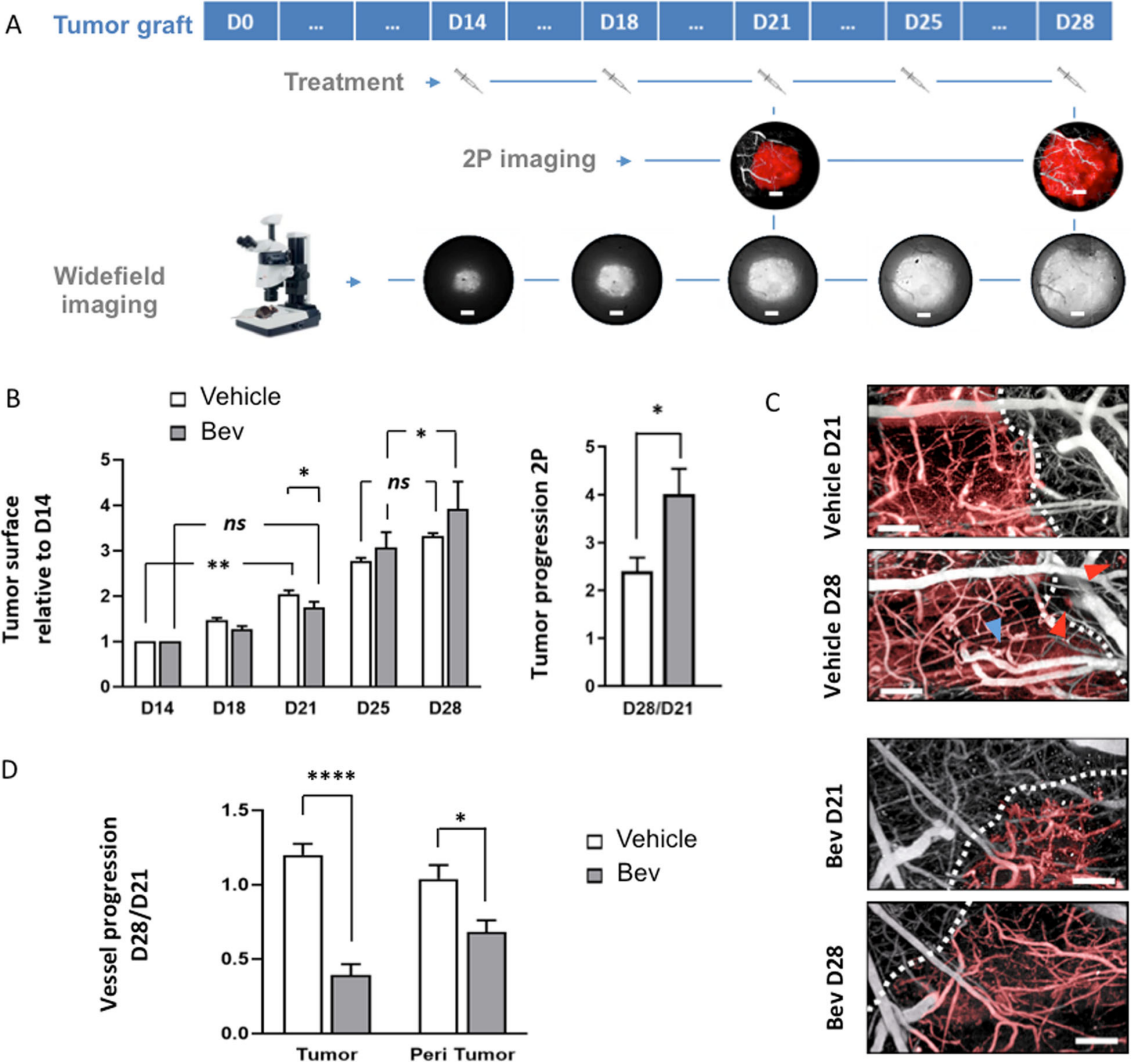
Effect of Bev treatment on tumor growth and blood vessel density over time. **a** Experimental timetable describing when intravenous treatment (vehicle or Bev) were performed after tumor graft (D0) as well as biphotonic (2P) and widefield acquisitions. Scale bars, 400 μm. **b** Evolution of the tumor growth in vehicle- (*n* = 4) or Bev (*n* = 3)-treated mice. Left panel, tumor diameters measured with a fluorescence widefield microscope and normalized by the size at the first day of treatment (D14) (*p < 0.05). Right panel, tumor volumes measured by in vivo two photon microscopy at D21 and D28 (*p < 0.05). ns, not significant. **c** In vivo 2P high-resolution images (3D projection, 150 μm depth) showing the micro-vascularization observed inside and around tumor at D21 and D28 after grafting for untreated (upper panel) or Bev-treated (lower panel) mice. Vessels in tumor area are outlined in red, the blue arrow shows an increase in blood vessel diameter and red ones indicate cells invading the adjacent healthy brain. Scale bar 100 μm. **d** Blood vessel density progression (D28 normalized on D21) measured thanks to in vivo 2P imaging for tumoral (****p < 0.0001) and peritumoral (*p < 0.05) zones for vehicle (*n* = 5) and Bev (*n* = 4) treated mice

The experimental timetable is summarized in Fig. 1a. Mice were imaged from day 14 until day 28 post-surgery. For time-lapse and cytometry experiments, animals were observed at day 21 and day 28.

#### Microscopy

Imaging was conducted essentially as reported [19]. Briefly, prior to each imaging session, mice were anesthetized and injected intravenously with 100 μL of a quantum dots (QDots) solution (Qtracker™ 705 Vascular Labels, ThermoFisher, 6 μg/100 μL in phosphate saline buffer, Sigma) and positioned on a stereotaxic frame allowing movements in the three-directions. Repositioning of the mice at each imaging session was realized using visual vascular landmarks.

We used a Zeiss LSM 780-MP two-photon microscope home modified to allow animal positioning below the 20X water immersion objective (1.0 NA) and coupled to a femtosecond pulsed infrared tunable laser (Chameleon Ultra 2, Coherent). Images were acquired using an excitation wavelength tuned at 920 nm to excite all fluorophores simultaneously. Signals were epicollected and separated by dichroic mirrors and filters on five independent non-descanned detectors. Gains and offsets were identical for all the detectors, except for the red channels whose gain was reduced by 30% to compensate for the strong expression of DsRed in tumor cells.

For CD11c-EYFP and LysM-EGFP cell recruitment experiments, images were typically acquired over a depth of 500 μm using 10μm steps. Laser power was linearly increased with depth. Z-stack images were acquired as mosaics (stitching mode) in order to cover the whole tumor surface with the peritumoral tissue that surrounds it.

For time-lapse experiments, stacks of images were acquired from 120 to 200 μm below the dura-matter with a z-step of 3 μm. The acquisition was realized over 3 h with time steps of 5 min. Ultra-fast time-lapse experiments were also realized from 150 to 180 μm below the dura-matter over a period of 20 min at 0.2 Hz repetition rate.

#### Data analysis

Spectral unmixing was first applied to raw 2P images (Zen software) and the mean cell density (number of cells/mm^3^) was calculated over tumoral volume using Imaris software v9.1 (Bitplane). The volume of each tumor was defined by creating a 3D mask using the surface tool from Imaris. Only cells present in the tumor mask were segmented according to the local intensity contrast. Cells found in blood vessels were easily excluded thanks to a 3D mask associated to the vasculature. Hence, to access the different cell densities, the reported number of CD11c-EYFP^+^, LysM-EGFP^+^/CD11c-EYFP^+^, and LysM-EGFP^+^ cells was reported over the tumor volume at each time point. Colocalization analyses were performed using Imaris software v9.1 (Bitplane) to identify LysM-EGFP+/CD11c-EYFP+ double-labeled cells.

Time-lapse z-stacks were processed using the open-source software Fiji and dedicated macros. Hyperstacks were first registered in 3D and cells of interest were manually tracked in 3D using the plugin MTrackJ [23].

### Other experiments

#### Cytometry

**Fig. 2:**
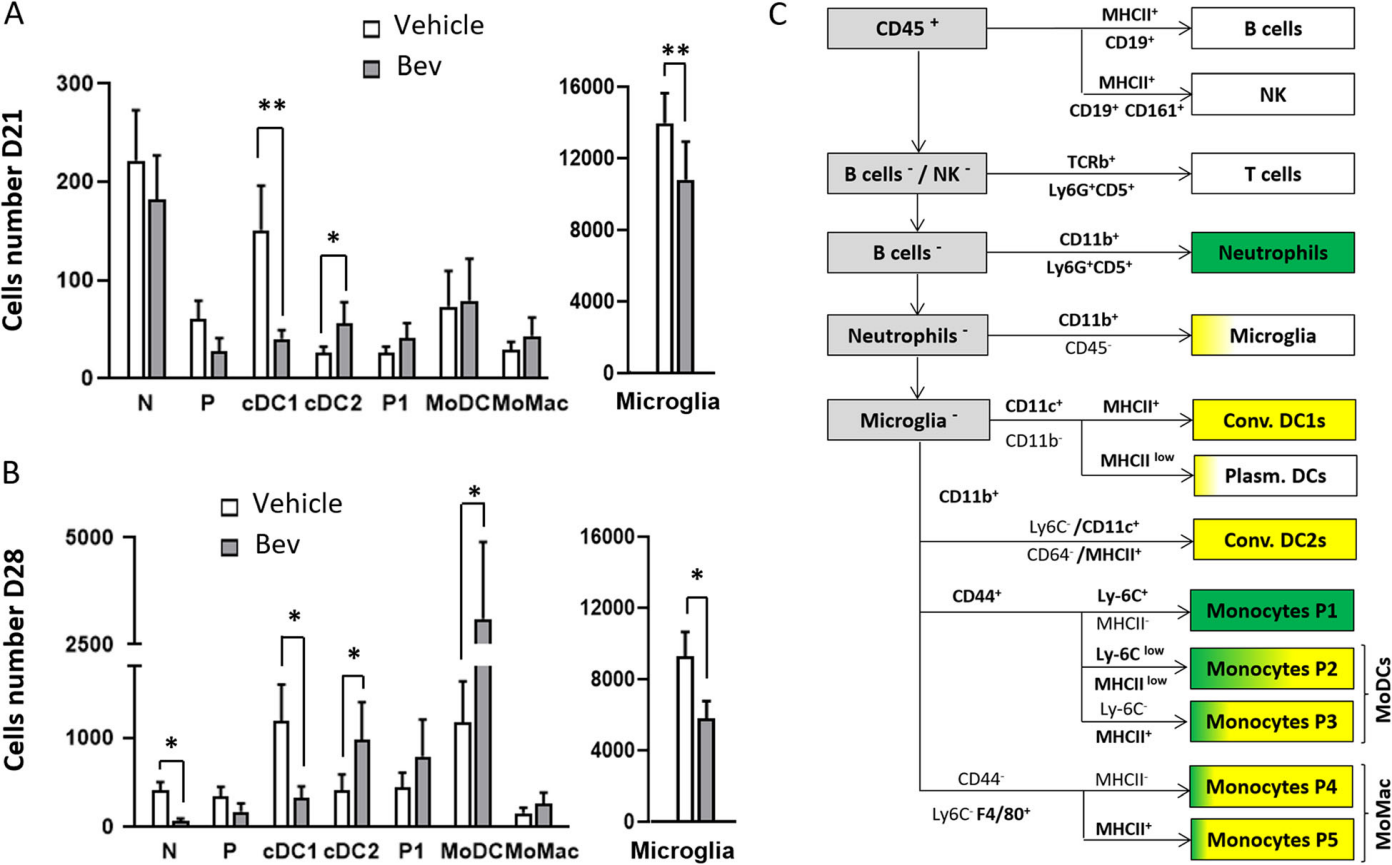
Multiparametric analysis of whole brain innate immune cells. Absolute numbers of neutrophils (N), dendritic cells DCs (P plasmocitoïd DC + conventional DC1 and DC2), monocytes/macrophages P1, moDC (P2/P3), moMac (P4, P5) cells as defined in Tamoutounour et al. [27] and Guilliams et al. [28]; (monocyte-derived DCs represent monocytes P2 and P3 whereas monocyte-derived macrophages represent monocytes P4 and P5) and microglia in the brain of vehicle or Bev-treated mice at D21 (*n* = 12 and 7) **a** and at D28 (*n* = 13 and 4) **b**; *p < 0.02. **c** Scheme of the gating strategy used for analyzing the cells contained within the brain. Percentage of cells expressing EGFP and/or EYFP in the cell subsets as well as their expression of MHCII

Animals were deeply anesthetized by an intraperitoneal injection of a mixture of xylazine/ketamine and were perfused by cardiac injection of 20 mL of PBS 1X. Brains were extracted, olfactory bulbs and cerebellum were discarded, and the cerebral hemisphere containing the tumor was excised. Cerebral tissues located below the corpus callosum including ventricles were removed. Then, tumor and surrounding healthy cerebral cortex including dura-mater were dissociated with DNAse I, Collagenase D (Roche Applied Science), and Collagenase V (Sigma) on a GentleMACS Octo Dissociator (Miltenyl Biotec). The suspension was then filtered while enzymatic reaction was blocked with EDTA. The cell suspension was centrifuged in an 80%/40% Percoll density gradient, rinsed then incubated 20 min with a mix of antibodies (see Additional file 8: Table S1) and labeled with Sytox Blue (Life technology). Acquisition was realized on a 5-laser BD LSRFortessa FACS equipment and data were extracted using the BD FACSDiva software. All acquisitions were done in a standardized way using application settings. The gating strategy used to extract immune cells had been described [19] and further illustrated in Fig. 2c indicating expression of fluorophores among cells subsets. Tumor cells were excluded on the basis of their DsRed fluorescence. The remaining CD45^+/low^ leukocytes tumor^−^ cells were further analyzed. The microglia, neutrophils, eosinophils, NK cells, and B cells were successively excluded. The remaining cells were separated on the basis of CD11c and CD11b into three populations. CD11c^−^CD11b^−^ cells correspond mainly to CD5^+^ T cells, CD11c^+^CD11b^−^ cells comprise pDC and cDC1, whereas CD11b^+^ cells comprise Ly-6C^−^ CD64^−^ CD11b^+^, cDC2, and CD44^+^ monocytes, and monocyte-derived DCs (moDC P2-P3) and CD44^−^monocyte-derived macrophages (moMac P4-P5).

#### Immunohistochemistry

Brains were extracted and fixed overnight in 4% paraformaldehyde (PFA). Then, they were cryoprotected by a 24h bath in 30% sucrose. The entire brains of Bev-treated (2) or control (2) mice were cut with a cryotome in 25-μm-thick coronal slices then permeabilized in a 0.5% Triton solution. A 1-hour blockage of the non-specific sites was realized (bovine serum albumin 2%, goat serum 2%, donkey serum 2%, FcR Blocking 1/10, Triton 0.1%) and primary antibodies were incubated overnight at 4 °C in PBS (rabbit anti-Iba1, 1/200, Wako (019–19,741); rat anti-MHCII, 1/50; rat anti-Ly6G, 1/50, BD Biosciences (clone 1A8), Secondary antibodies (goat anti-rabbit conjugated to Dylight405, 1/100, Thermo (35551); donkey anti-rat conjugated to Cy5, 1/100, Jackson Immunoresearch (712–175-150) were incubated 1 h 30 at room temperature. Slices were then mounted with Vectashield. Slices were washed in PBS in between the different steps. Observations were performed on a Zeiss LSM780 confocal microscope in the spectral mode, using 405, 488, 514, and 633 nm excitation wavelength. Twelve to 15 slices were analyzed for each combination of staining. The intra-tumoral cells of interest were counted on each section, and their number was systematically normalized to the surface of the tumor.

#### Brain clearing

We performed a cubic clearing of whole-brains as described by Susaki et al. [24]*.* Mice were perfused with 10 mL PBS (pH 7.4) (Thermofisher Scientific), followed by 25 mL of cold PFA 4% (pH 7.4) (Electron Microscopy Sciences). Brains were removed and post-fixed in 10 mL PFA 4% at 4 °C for 18–24 h. Brains were washed in 10 mL PBS twice for 2 h at room temperature. After washes, they were immersed in 5 mL of CUBIC1 (25% Urea, 25% Quadrol-80%, 15% Triton X100, qsp H2O, Sigma-Aldrich) diluted (V/V) with mQ water, shaken at 5 rpm at 37 °C for 3–6 h. Then, the solution was removed and samples were immersed in CUBIC1 for 8 days, at 37 °C. CUBIC1 solution was replaced every 2 days. Samples were washed in 20 mL PBS at room temperature 3 times for 2 h. Next, brains were immersed in 5 mL of CUBIC2 (25% Urea, 50% sucrose, 10% Triethanolamine, qsp mQ water, Sigma-Aldrich) diluted with PBS (V/V) and shaken for 6–24 h at 37 °C. The solution was removed and replaced by CUBIC2 for 24 h.

#### Statistical analysis

All data are expressed as mean ± SEM. Mann-Whitney and two-way ANOVA were used to test differences in between time points. *p* < 0.05 was used as a criterion for significance (*). All statistical analyses were performed with Microsoft Excel and Graphpad Prism.

## Results

### Effect of Bev treatment on tumor size and vessel density

We used our previously established syngenic orthotopic GBM model in immunocompetent C57BL/6 Thy1-CFP//LysM-EGFP//CD11c-EYFP triple-transgenic animals [19] that recapitulates the typical features of the GBM tumor and is suitable for intravital two-photon imaging analyses. This model employs the stereotaxic injection of a spheroid of fluorescent GL261 glioma cells stably expressing DsRed into the superficial layers of the parietal cortex (approximately 250 μm deep). The dura-mater is sealed with a Sephadex hemi-bead and the bone above the injection site is replaced by a glass window (4–5 mm in diameter). The cranial window allows a non-invasive follow-up of the events throughout the disease progression. Tumor growth was measured by fluorescence macroscopy every 3–4 days from day 14 until day 28, the final stage of the disease at which all mice lost weight and some died.

Grafted mice were separated in two batches: control mice (*n* = 7) received only the vehicle (PBS) whereas 7 other mice were treated with Bev (25 mg/kg), intravenous, at days 14, 18, 21, 25, and 28 (Fig. 1a). We verified that at day 14 before Bev treatment, the mean tumor area was similar for both groups (Additional file 1: Figure S1A).

As already described [17, 25, 26], we observed that this high clinically relevant dose induced an uncoupling of the antitumoral and the antivascular effects of Bev. Tumor expansion fits with an exponential curve with slow growth until day 21 after grafting followed by rapid expansion afterwards (Fig. 1b). Bev transiently slowed down tumor growth rate in between day 14 and day 21 after grafting (Fig. 1b, left panel). After day 21, the tumor escaped the treatment since it grew even faster than the control tumor when tumor progression was compared between day 21 and day 28. This data was also confirmed by comparing progression of tumor volume between day 21 and day 28 for each mouse using two-photon intravital microscopy (Fig. 1b, right panel). Recurrent two-photon imaging of GL261-DsRed grafted mice receiving an intravenous injection of QDots 705 to highlight blood vessels revealed the effect of Bev treatment on vessel normalization. The tumor elicited vascular remodeling in its core (Fig. 1c), while individual tumor cells invading the adjacent healthy brain parenchyma can be observed. The effect of Bev treatment on vessel density was striking, and we observed a very significant and persistent effect of Bev on vessel density especially inside the tumor also detectable but less pronounced in the peritumoral area (Fig. 1d, Additional file 1: Figure S1B). A laminin immunostaining on tumor sections from treated and untreated animals also confirmed the decrease in vessel density (Additional file 1: Figure S1C).

### Phenotyping innate immune responses during Bev treatment

To document the degree of heterogeneity found in the innate immune cell populations during Bev treatment, we performed multiparametric FACS analyses [27]. Throughout this manuscript, we employ the ontogeny-based classification/nomenclature as proposed by Guilliams et al. [28]. We collected and dissociated tumors and neighboring cortex tissues of PBS perfused individual mice, treated or not with Bev, and taken at the beginning (day 21) and after (day 28) the massive tumor invasion by circulating leucocytes. In a previous study [19], we reported that at day 21, the brain immune profile of tumor-bearing animals, determined by FACS was essentially the same for control and sham-operated animals, both in terms of cell numbers and fluorescent protein expression. Here, in untreated tumor-bearing mice, we observe in between day 21 and day 28, a dramatic change of immune profile. Indeed, at day 28 when compared to day 21, the representation of CD45^low^/CD11b^+^ population, corresponding to microglial cells, decreases (compare Fig. 2a and b, right**)** concomitantly with the fivefold increase in the number of CD45^+^ infiltrating cells. Microglial cells outnumbered infiltrated cells by 45-fold at day 21 but are only 2.7-fold more numerous at day 28 (Fig. 2a and b).

We then examined whether and how Bev treatment modifies recruited innate cell composition and function by comparing treated and untreated tumor-bearing mice at day 21 and day 28 (Fig. 2a and b). To this end, we performed as previously described [19, 29]*,* successive gating on 13 membrane proteins in addition to CD45 and CD11b (Additional file 8: Table S1), to untangle and quantify the corresponding populations of innate immune cells.

At day 21, the dominant population invading the brain parenchyma and recovered in the brain cell suspension was neutrophils, which outnumbered dendritic cells (DCs) and monocytes/macrophages (P1–P5) in both in Bev-treated and untreated mice (Fig. 2a, upper panel). However, Bev treatment significantly influences subtypes of DCs with a lower number of Conventional DC1 (cDC1) at the expense of CD11b^+^ DCs (cDC2). We also observed a trend towards a higher recruitment of monocytes at stage P1 of maturation. The precursor P1 subpopulation differentiated into two populations: a predominant CD44^+^/CCR2^+^ population composed of P2 and P3 monocyte-derived DCs (moDC) and into a smaller CCR2^−^/CD44^−^ population composed of P4 and P5 macrophages (moMac). At day 21, no significant effect could be detected on moDC or moMac. At day 28, neutrophils represented a minor population significantly decreased by Bev treatment (Additional file 1: Figure S1D). Importantly, the overall number of monocytes and DCs dramatically increased (Fig. 2b, lower panel). It is noteworthy that one third of the total DCs present in the brain during tumor progression were moDCs (24% at day 21, 37% at day 28) whereas moMac remained a minor subset in treated or untreated mice. In Bev-treated mice, the number of P1 monocytes and their moDC progeny (P2/3) was increased by approximately twofold for each of the subpopulations. We observed a high variability between individual treated mice particularly in the P2 population (not shown), which can be explained by the fact that this population is transient. The effect of Bev on cDC2 and cDC1s subsets already observed at day 21 was maintained. cDC2 were twofold more numerous in Bev-treated mice, whereas the number of cDC1s was approximately threefold lower than in non-treated mice. The decrease in microglial cell number in treated mice already observed at day 21 was also maintained.

Finally, we evaluated the expression of LysM-EGFP and CD11c-EYFP fluorescence in each of the populations (Fig. 2c). The percentage of cells expressing the fluorophore in a given subset did not significantly vary depending on whether mice received Bev or not. All neutrophils and monocytes (stage P1) were EGFP^+^. Monocytes acquired expression of CD11c during their transition towards more mature stages and the moDC (P2/P3 population) expressed both EGFP and EYFP. Approximately 20% of the overall microglia population expressed EYFP. cDC 1 and 2 populations both expressed EYFP whereas the pDC are mainly not labeled.

### Imaging tumor and LysM-EGFP^+^ and CD11c-EYFP^+^ cell dynamics distribution during progression of GBM in control or Bev-treated mice

**Fig. 3:**
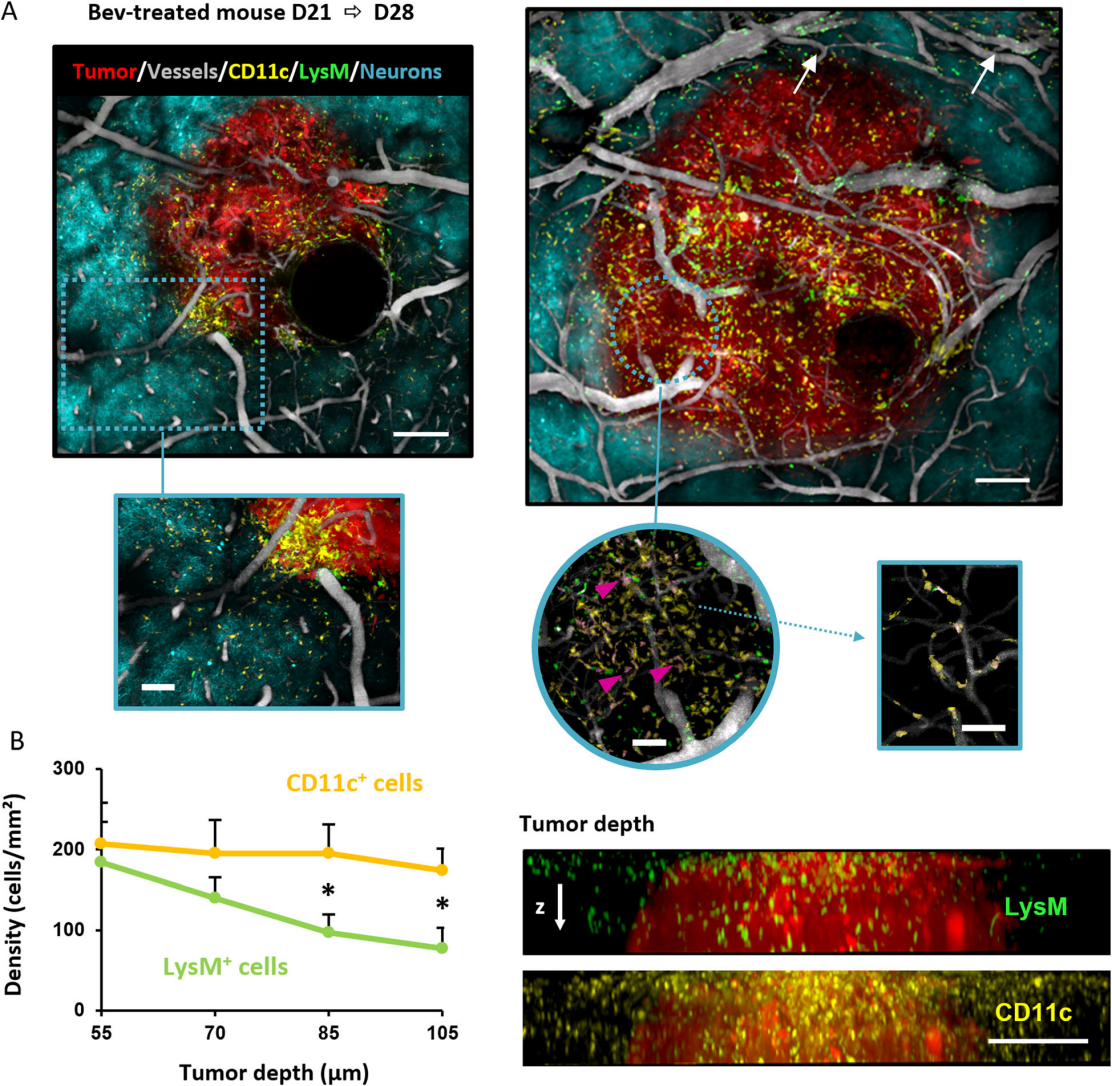
Spatial distribution of fluorescent cells relative to the tumor microenvironment. **a** Five color intravital 2P microscopy of a Bev-treated triple transgenic mouse at D21 (upper left) and D28 (upper right, arrows show the LysM-EGFP^+^ cells present in the vessels). Maximal intensity projection over 150 μm depth. Scale bar 200 μm. Zoom in of the outlined regions of interest from D21 (lower left, scale bar 100 μm) and D28 (lower middle and right, scale bar 50 μm). The cells colored in pink refer to double-labeled cells (pink arrows) (CD11c-EYFP^+^/LysM-EGFP^+^) inside the tumor. **b** Graph showing the density of CD11c-EYFP^+^ (yellow) and LysM-EGFP^+^ (green) cells as a function of tumor depth for Bev-treated mice. *p = 0.03. Images at D21 of a Bev-treated mouse showing the distribution of LysM-EGFP^+^ cells (green) and CD11c-EYFP^+^ cells (yellow) as a function of tumor depth (150 μm), starting from the top of the tumor (right panel, scale bar 200 μm)

Each mouse was repeatedly imaged by two-photon on days 21 and 28 after grafting. In both Bev-treated and untreated animals, the increase in tumor sizes was readily observable as well as different densities of LysM-EGFP^+^ and CD11c-EYFP^+^ cells between day 21 and day 28 for each individual animal (Fig. 3a upper panel). Whatever the condition or time post-graft, CD11c-EYFP^+^ cells were never observed in the circulation. LysM-EGFP^+^ cells were observed in tumor blood vessels and the tumor microenvironment (Additional file 2). In the tumor, they were closely associated with or at near vicinity of blood vessels (Fig. 3a lower left and right panel). We also observed double-labeled LysM-EGFP^+^/CD11c-EYFP^+^ cells identified as P2/P3 moDC (see above) inside the tumor (Fig. 3a, lower middle panel). More noticeable was the localization of the fluorescent populations with respect to the position of the tumor at a different time after grafting. At day 21, LysM-EGFP^+^ cells were more numerous on the top of the tumor near the meninges as a gradient could be observed from the periphery towards the parenchyma, whereas CD11c-EYFP^+^ cells were more evenly distributed inside or in peritumoral areas (Fig. 3b). The spatial distribution of fluorescent cells between Bev-treated and untreated mice does not differ. However, a striking difference was observed between Bev-treated and untreated mice, as a high density of LysM-EGFP^+^ cells could be seen in Bev-treated animals.

**Fig. 4:**
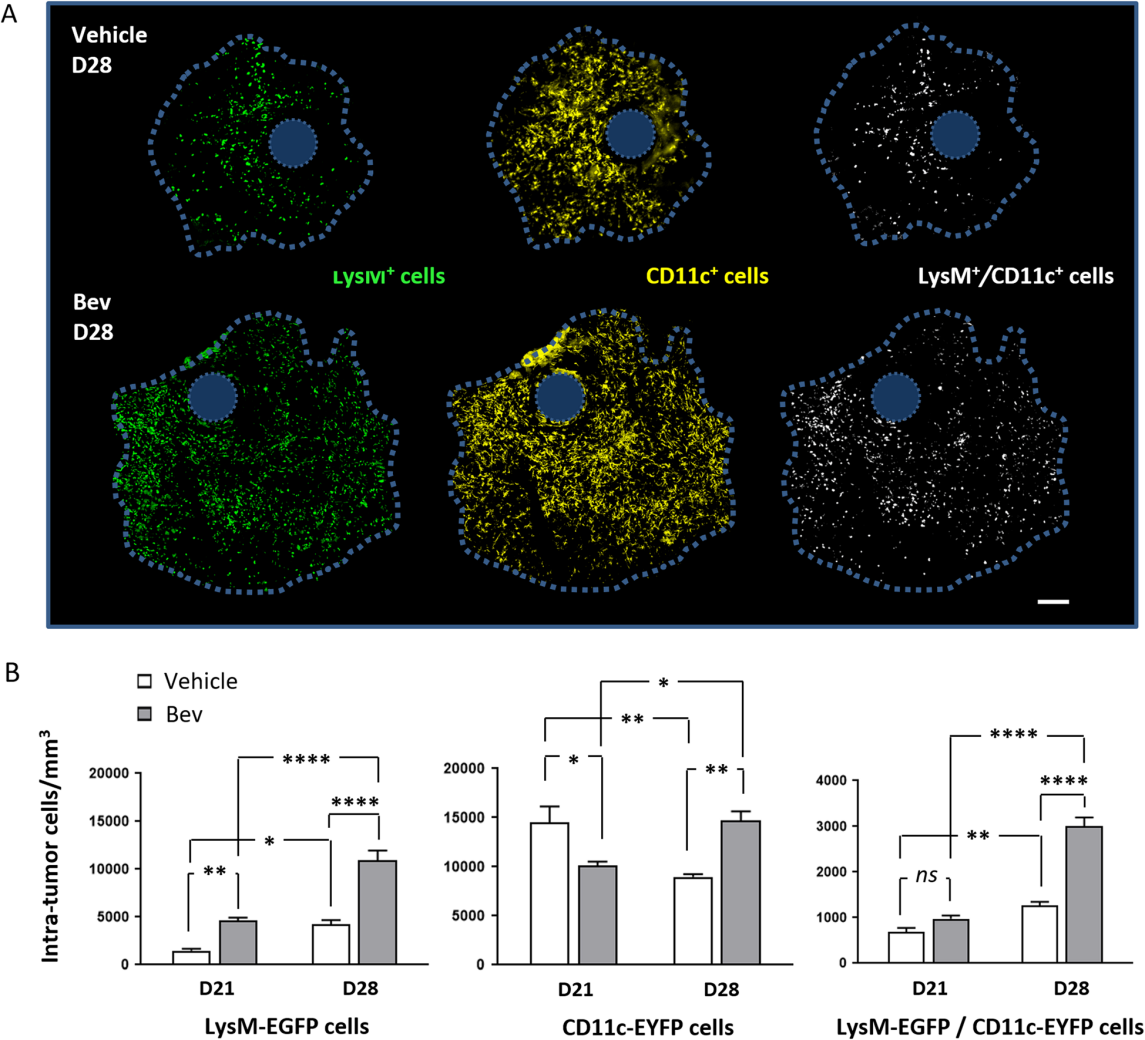
Quantification over time of intra-tumoral fluorescent cells. **a** Maximal intensity projection (100 μm z-stack) of LysM-EGFP^+^, CD11c-EYFP^+^, and LysM-EGFP^+^/CD11c-EYFP^+^ cells inside tumor of untreated (upper) and Bev-treated mice (lower) at D28. The blue dot represents the Sephadex hemi-bead location. Scale bar 200 μm. **b** LysM-EGFP^+^, CD11c-EYFP^+^, and LysM-EGFP^+^/CD11c-EYFP^+^ cells were counted as described in the method section at D21 and D28 in control (*n* = 4) and Bev-treated (*n* = 4) tumor-bearing mice (*p < 0.02) (**p < 0.008) (****p < 0.0001). ns, not significant

To obtain quantitative data for each mouse individually, we counted and determined the mean densities of LysM-EGFP^+^, CD11c-EYFP^+^, and double-labeled LysM-EGFP^+^/CD11c-EYFP^+^ cells over a volume of 100 μm depth inside the tumor for Bev-treated and untreated mice at day 21 and day 28. Each region of interest covered the entire surface of the tumor (Fig. 4).

Clearly, fluorescent cells were not homogeneously distributed on the region of interest and sometimes grouped in clusters (Fig. 4a). When the data were pooled for all the animals for a given condition, it appeared that inside the tumor both at days 21 and 28 (Fig. 4b**,** left panel), the density of LysM-EGFP^+^ cells was significantly higher in Bev-treated animals (i.e., mean density 4 × 10^3^ cells/mm^3^ for vehicle group *vs* 11 × 10^3^ cells/mm^3^ for treated group at day 21). Importantly, as supported by FACS data and imaging observations, at day 21 and day 28, Bev treatment appears to double the mean density of LysM-EGFP^+^ cells compared to untreated animals (Fig. 4b, left panel). By performing in vivo time-lapse recordings, we also noticed that the number of LysM-EGFP^+^ cells traveling in blood vessels was strikingly higher in Bev-treated mice (100 cells/min) compared to control mice (26 cells/min) (Additional file 2, Additional file 4: Video S1 and Additional file 5: Video S2).

The density of LysM-EGFP^+^ and that of their LysM-EGFP^+^/CD11c-EYFP^+^ (moDC) progeny significantly increased with time inside the tumor both in untreated and treated mice. Bev treatment very significantly increased the double-labeled subset when compared to untreated tumors at day 28 (Fig. 4b, right panel). The density of CD11c-EYFP^+^ cells significantly varied depending on time and treatment. At day 21, their density was higher in untreated mice. At day 28, whereas their density decreased in untreated mice (14 × 10^3^ at day 21 *vs* 9 × 10^3^ cells/mm^3^ at day 28), it increased in Bev-treated animals (10 × 10^3^ at day 21 *vs* 14.5 × 10^3^ cells/mm^3^) (Fig. 4b, middle panel).

### Quantitative analysis of multicolor immunostained sagittal brain slices of GBM control and Bev-treated mice

Observations by two-photon imaging revealed a heterogeneous distribution of fluorescent cells inside the tumor and in the parenchyma but technical limitations prevent imaging the whole tumor and its surrounding in its later stage. The expression profiles of EGFP and EYFP determined by FACS **(**Fig. 2c) indicated that whereas EGFP is essentially associated to P1 monocytes at a later stage, EYFP expression is shared by several cell subsets. These are approximately 20% of the microglial population as well as all the cDC1, cDC2. To gain a better understanding of the patterned distribution of fluorescence in the tumor and peritumoral area and possible function of EYFP^+^ cells, we combined observation of EGFP and EYFP with immunostaining on tissue sections.

**Fig. 5:**
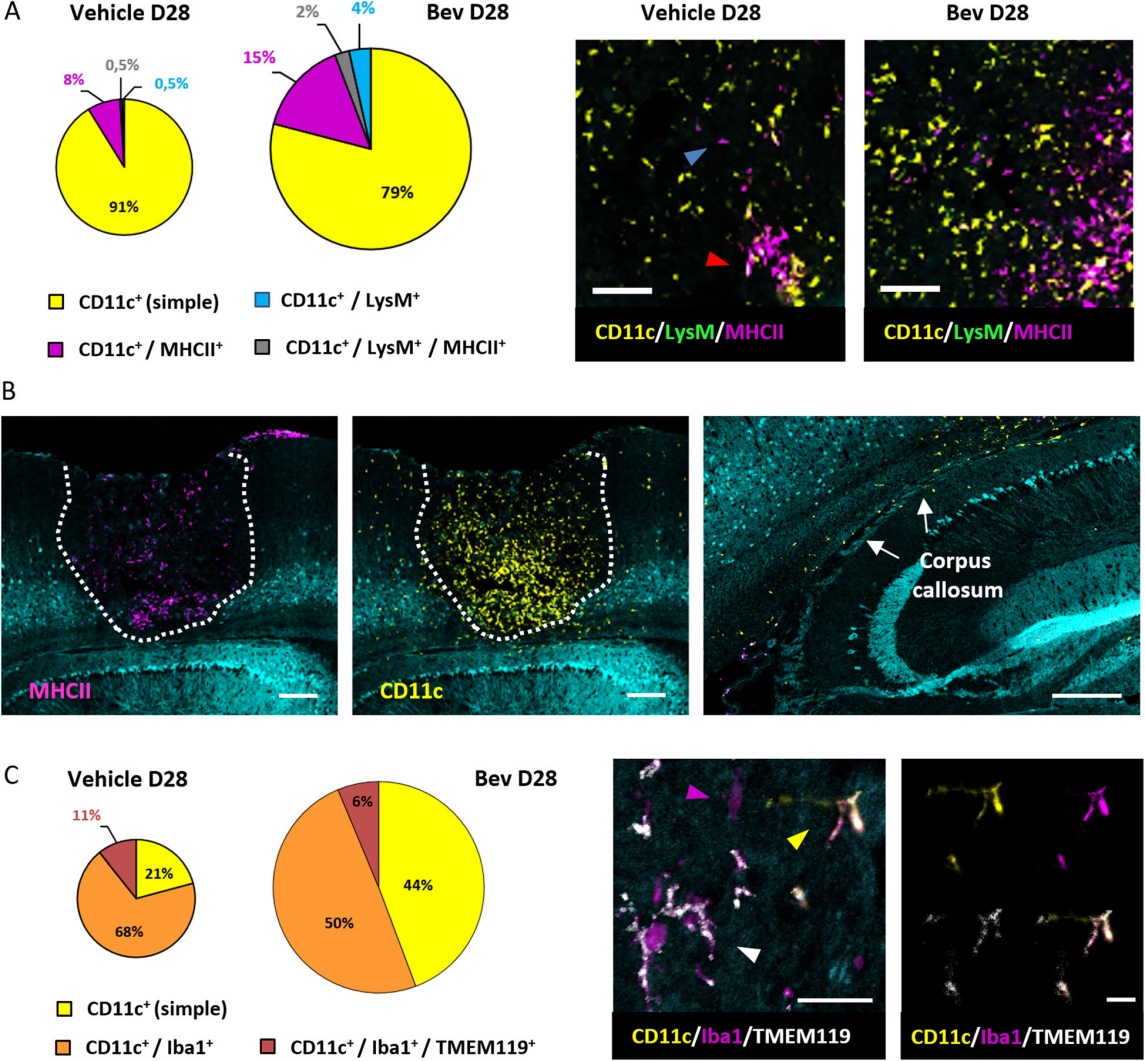
Phenotypic characterization of immune cell populations in GBM-bearing mice by multicolor immunochemistry. **a**, **c** The surface of the pie charts is proportional to the absolute cell number in the specified population after normalization for the tumor area (see for details Additional file 3**:** Figure S3a). **a** Percentages of CD11c-EYFP^+^ single-labeled (yellow), CD11c-EYFP^+^/MHCII^+^ (magenta), CD11c-EYFP^+^/LysM-EGFP^+^ (blue), and CD11c-EYFP^+^/LysM-EGFP^+^/MHCII^+^ (gray) cells among the CD11c-EYFP^+^ population found in intra-tumoral zones at D28 (left) and examples of multicolor immunostaining of CD11c-EYFP^+^ (yellow) and MHCII^+^ (magenta) cells in sagittal sections of untreated (vehicle) and Bev-treated mice at D28. Red arrow shows double-labeled CD11c-EYFP^+^/MHCII^+^ cells and blue arrow MHCII^+^ single-labeled cells. **b** Dotted-lines highlight tumor margins. Note that CD11c-EYFP^+^ and MHCII^+^ cells are denser in the deepest part of the tumor. Right panel, Fluorescent cells can also be seen along the corpus callosum. Scale bars 200 μm. **c** Percentages of CD11c-EYFP^+^ single-labeled (yellow), CD11c-EYFP^+^/Iba1^+^ (orange), and CD11c-EYFP^+^/Iba1^+^/TMEM119^+^ (red) cells found in intra-tumoral areas of untreated (vehicle) and Bev-treated mice at D28. **d** Microglia is Iba1^+^/TMEM119^+^ (white arrow) and an activated microglia subset is CD11c-EYFP^+^/Iba1^+^/TMEM119^+^ (yellow arrow). Note that Iba1 is also expressed by cells other than microglia (magenta arrow). *n* = 2 mice/condition. Scale bar 20 μm. Right panel, triple-labeled microglial cell CD11c-EYFP^+^/Iba1^+^/TMEM119^+^. Scale bar 10 μm

We focused on co-expression of the EYFP fluorescence with MHCII, a molecule expressed by antigen presenting cells, and TMEM119, a transmembrane protein, reported to be a highly specific microglia marker not expressed by macrophages or other immune or neural cell types [30] therefore more specific than Iba1. Quantitative analysis of multicolor immunostained sagittal brain slices at day 28, when the difference of cell densities is the highest between treated and untreated animals, showed that most (approximately 90%) of the intra-tumoral CD11c-EYFP^+^ were not expressing detectable levels of MHCII in the control group. In treated animals, approximately 20% of the fluorescent cells strongly expressed MHCII and among these 2% representing one third of the moDC co-expressing EGFP and EYFP, and a larger proportion (15%) of other fluorescent DCs (cDC1 and cDC2) (Fig. 5a, Additional file 3: Figure S3A). Interestingly, the observation of stained sections revealed that CD11c-EYFP^+^/MHCII^+^ cells were denser in the deepest part of the tumor. It also revealed some of these fluorescent cells along the corpus callosum (Fig. 5b). Moreover, CD11c-EYFP^+^/MHCII^+^ cells were observed in the choroid plexus, together with rare MHCII^+^ cells not expressing CD11c (Additional file 3: Figure S3B). By contrast, the non-expressing MHCII cells were observed in the higher part of the tumor and in the meninges, thus revealing different localization of cell subsets of different maturation states or function (Fig. 5b).

In the tumor core, a large proportion of the EYFP^+^ cells were expressing Iba1 (70% in control and 50% in treated mice) but not TMEM119 (Fig. 5c). Among them, only 10% in untreated and 6% in Bev-treated mice were Iba1^+^ and TMEM119^+^, which supported their microglial identity. However, a high density of such cells could be observed in the neighboring parenchyma in untreated mice (Additional file 3: Figure S3C right panel). CD11c/TMEM119/Iba1^+^ cells identified only a microglia subset in a particular activated state. This data suggests that Bev might influence not only the number of microglial cells as shown by FACS but also their activation state.

Overall, these experiments support in vivo imaging quantifications and FACS data. Importantly, they also revealed a so far undescribed spatial distribution of cell subsets.

### 3D observation of cell localization after tissue clearing

**Fig. 6:**
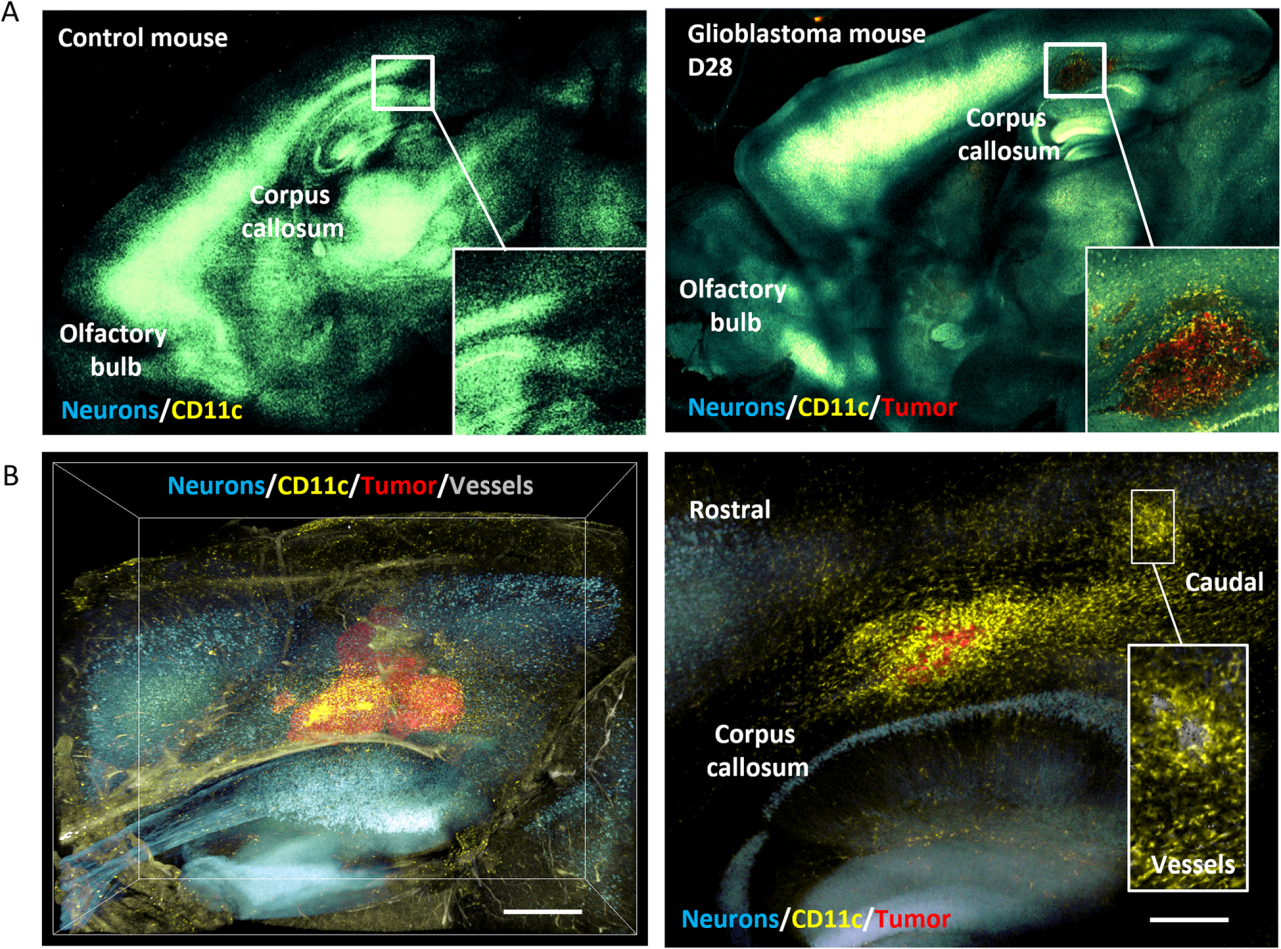
Distribution of immune cells in mouse physiological brain and GBM-bearing brain. Cubic-clearing treatment and 2P imaging of whole-brains of Thy1-CFP//LysM-EGFP//CD11c-EYFP mice. **a** 2P imaging of a half-cleared brain. Left panel, normal brain, no LysM-EGFP^+^ (not shown) or CD11c-EYFP^+^ cells could be detected. Right panel, tumor-bearing mouse at D28. Enlargement shows CD11c-EYFP^+^ cells traveling along the corpus callosum. **b** High-resolution five-color 2P imaging of a cleared GBM-bearing brain. Left panel, 3D sagittal view of the tumor showing distribution of fluorescent cells towards the tumor. Scale bar 500 μm. Right panel, view of fluorescent cells located below the tumor, around blood vessel, and along the corpus callosum. Scale bar 300 μm

The observations by two-photon and on tissue sections strongly suggested two routes of innate cells entry and/or exit to the parenchyma, namely meninges and corpus callosum towards choroid plexus. To further confirm these observations, we performed tissue clearing using the CUBIC technique, on Thy1-CFP//LysM-EGFP//CD11c-EYFP triple transgenic mice in which axons are CFP fluorescent, which confirmed that EYFP and EGFP cells were absent in healthy brain (Fig. 6a, left panel). In GBM bearing mice at day 28, a high density of CD11c-EYFP^+^ cells was observed in the corpus callosum (Fig. 6a, right panel). We also noticed at this stage that CD11c-EYFP^+^ cells distributed in a gradient fashion from the meninges to the inner parenchyma near the tumor (Fig. 6b) suggesting a maturation of EGFP monocytes in EYFP-moDC as they migrate towards the tumor core.

### Dynamic intravital imaging to gain insight into cell interactions

Innate cells, in particular monocytes, are continually trafficking in the tumor microenvironment. During this time they may undergo immuno-education leading to their transformation/maturation. This process could occur secondary to cell-cell contact between naïve monocytes and GBM cells or, alternatively, due to exposure to the cytokine-rich tumor environment. Our above observations suggest that this environment differs between Bev-treated and untreated mice as densities and phenotypes of DCs subpopulations differ.

**Fig. 7:**
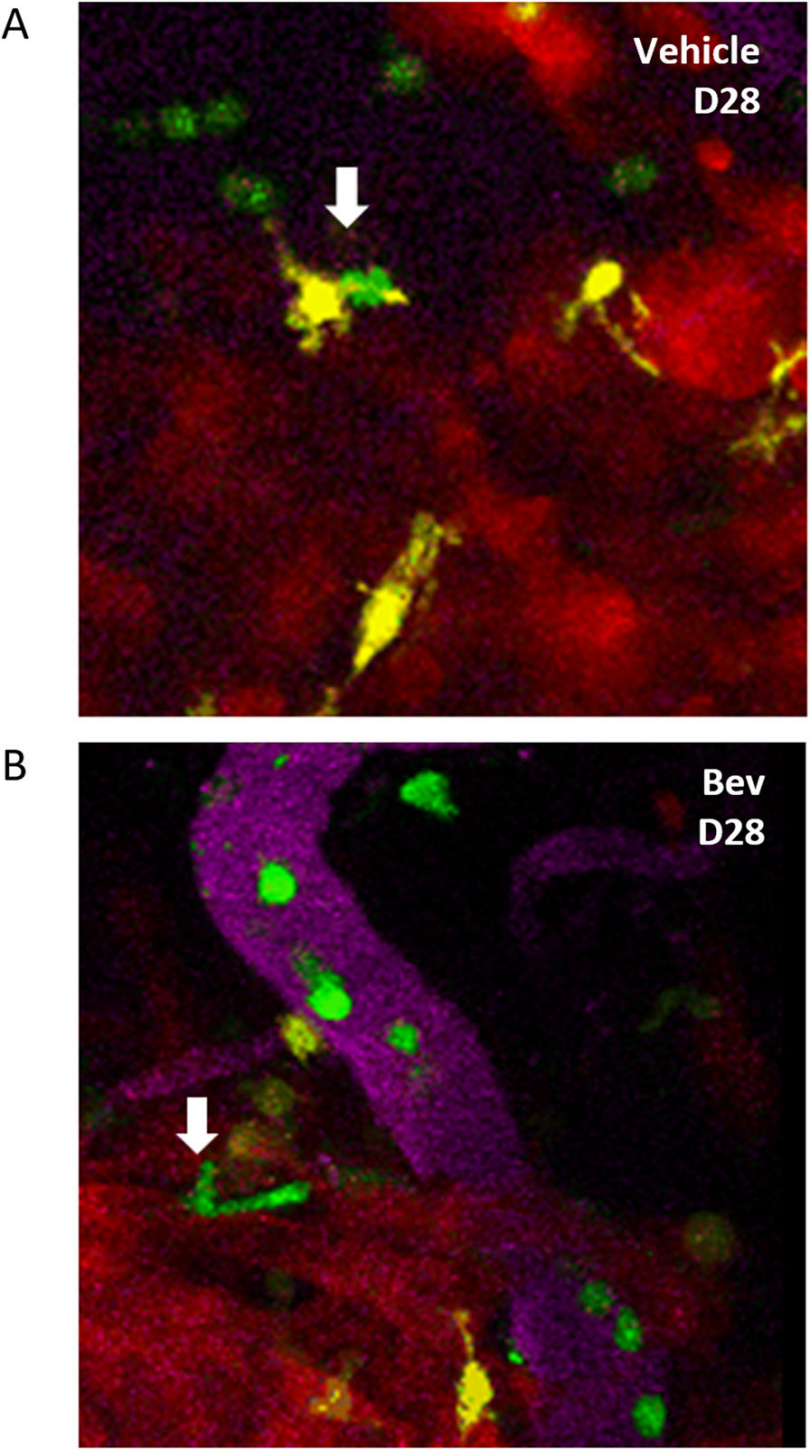
**a** Two-photon images illustrating representative physical interactions occurring between a LysM-EGFP^+^ monocyte and a CD11c-EYFP^+^ cell in the tumor core and **b** of a LysM-EGFP^+^ monocyte with tumor cell

We took advantage of two-photon spectral imaging to observe the behavior of our fluorescent cells by performing time-lapse recordings. In both treated and untreated mice, we observed frequent contacts between LysM-EGFP^+^ and CD11c-EYFP^+^ cells (Fig. 7a) as shown in real time in Additional file 6: Video S3 and in Bev-treated mice frequent contacts of LysM-EGFP^+^ cells with tumors (Fig. 7b) and Video S4 (Additional file 7: Video S4).

## Discussion

VEGF is an important factor in tumor vascularization and used as target for anti-angiogenic treatment strategies in GBM. Based on promising phase II trials, Bev, an anti-VEGF monoclonal antibody, was approved by the Food and Drug Administration as monotherapy for GBM. However, three randomized phase III trials showed that Bev, despite extending progression-free survival failed to prolong patients’ overall survival [6]. The underlying mechanisms of acquired resistance to anti-angiogenic therapy remain obscure. In part, this is a result of the limited understanding of the effects of anti-VEGF therapy on the microenvironment of the tumor. Detailed information on how Bev affects GBM is important not only to understand the success or failure of such treatment, but also to provide educated advice on how combination therapies should be optimized.

In a recent study using the GL261 GBM mouse model, Turkowski *et al*. [8] reported that VEGF is a modulator of the innate immune response with suppressive effects on the immunologic and pro-angiogenic function of microglia/macrophages. They showed that high level of VEGF expressed by engineered tumor cells led to threefold enlarged tumor volumes and a pronounced remodeling of the vascular structure along with a reduced infiltration of microglia/macrophages by approximately 50%. Here, we investigated the reverse situation by depleting VEGF. We used multicolor fluorescent reporter mice and in vivo spectral two-photon microscopy to gain insight on the effects of the depletion at two critical stages of tumor development in individual animals. These observations were combined with multiparametric cytometry and immunohistochemistry to link information on cell distribution with precise phenotypes and frequencies of innate cell subsets with a focus on DCs. In addition, to describe dynamic effects on cell subsets, our approaches uncovered so far undescribed spatio-temporal distributions of innate cell subpopulations in the tumor microenvironment and potential routes entering and/or exiting the brain parenchyma.

We started Bev treatment at day 14 after tumor grafting. As expected and already described [17, 19, 25], Bev persistently normalized intra-tumoral vessels and transiently slowed down tumor growth rate but the tumor then escaped the treatment as shown by comparing progression in between day 21 and day 28. Nonetheless, the effect on tumor size of VEGF blockade is less drastic than that of VEGF over-expression [8]. Recurrent two-photon imaging of GL261-DsRed grafted mice after injection of QDots 705 to highlight functional blood vessels also revealed a persistent treatment effect on vessel density and tortuosity especially inside the tumor. These observations confirmed observations we made in a U87-MG model [17] that tumor growth can be sustained without an increase in blood vessel density suggesting that GBM growth is rather governed either by vessels and/or stromal properties.

Intravital imaging and time-lapse recordings of fluorescent cells (Additional files 1 and 2: Videos S1 and S2) revealed their high association to the tumor vasculature. Importantly, we observed that under Bev treatment, the number of LysM-EGFP^+^ cells trafficking inside the vessels was significantly higher than in untreated mice. A possibility is that anti-VEGF treatment results in an increased blood flow that might occur during the tumor vessel normalization window [31]. At both day 21 and day 28 LysM-EGFP^+^ density in the tumor and its environment is more than twofold higher in Bev-treated mice than in controls. We cannot infer, however, that a change in the properties of tumor blood vessels and their normalization induced by Bev [32] is directly related to this increase. Indeed, both in control and treated mice, we never observed extravasation of these cells from tumor blood vessels. Interestingly, imaging revealed a gradient of LysM-EGFP^+^ cells from the meninges towards the tumor core. This supports the view that meningeal vessels are potential gateways for immune cells recruited into the brain [33]. In addition, a recent publication [34] points to a direct local interaction between the brain and the skull bone marrow through the meninges. The authors determined the origin of leukocytes that were recruited to cerebral inflamed tissues and found that skull bone marrow myeloid cells migrate towards the inflamed brain through microscopic channels that directly connect the skull marrow cavities with the dura. The spatial distribution revealed by brain clearing (Fig. 6b) would also support such an origin as we observed a gradient of fluorescent cells in the parenchyma, potentially recruited towards the tumor.

The FACS data (Fig. 2a and b) indicated that at an early stage (day 21) neutrophils outnumbered monocytes among LysM-EGFP^+^ cells. At day 28, LysM-EGFP^+^ cells are essentially monocytes at stage P1. All our data (FACS, imaging) converge to support a decrease in recruitment of neutrophils and the increase of the recruitment of monocytes at stage P1 as a consequence of VEGF blockade. Our previous observations [19, 29] indicated that monocytes differentiate in situ in the pathologic CNS into either moDC (P2–P3) or macrophages (moMac P4–P5). MoMac are very scarce and we could not determine whether Bev significantly modifies their levels. During these dynamic differentiation processes monocytes start to express CD11c-EYFP and moDC are co-expressing the two fluorophores. In agreement with the increased recruitment of monocytes, and in support of this differentiation process, VEGF blockade increases the density of moDC with twice as many intra-tumoral double-labeled cells in Bev-treated compared to untreated mice (Fig. 4, right panel).

Taken together, our data indicated that in our model, LysM-EGFP^+^ cells recruitment starts at a rather precise time during the development of the tumor (around days 20–22 after tumor grafting) with recruitment of neutrophils, which then stopped, concomitant or followed by recruitment of monocytes that continues thereafter. The progressive wavelike shift in the phenotypes of infiltrated monocytes corresponds to the refractoriness of the tumor to treatment. As already reported [19], after day 21, microglia representation significantly decreases among the innate cell subsets and the EYFP^+^ population. This dynamic change is in agreement with the view that resident microglia rather than peripheral leucocytes promote vascularization in brain tumors [11]. Bev appears to further decrease the number of activated microglial cells in the brain both at an early stage (day 21) and later stage (day 28) of tumor development (Fig. 2). Whereas FACS analysis concerned the tumor and the surrounding brain parenchyma, the observation is further supported by immunolabeling of intra-tumoral CD11c-EYFP^+^/TMEM119^+^/Iba1^+^ cells at day 28 (Fig. 5c, Additional file 3**:** Figure S3A).

In their study, Turkowski *et al*. [8] observed that VEGF over-expression resulted in a reduced expression of microglia/macrophages by approximately 50%, whereas several published data report an increase in the amount of these cells elicited by anti-angiogenic treatments. It should be pointed out that most of the published data are almost exclusively performed on the entire myeloid cell fraction because the lack of lineage markers prevented their classification into subsets. The authors infer that the increase in the amount of innate immune cells conduces to resistance development in tumors but pro- and antitumoral characteristics were not assigned to the evident subpopulations [14, 15].

In an effort towards this goal, we deciphered precisely the key populations, i.e., DC subsets and controlling immune responses, that are affected by VEGF blockade. We report that the blockade elicited changes in the ratios of the different subsets present during the escape phase of the tumor. At day 28 among the EYFP^+^ subsets, besides the 6–7% of cells identified as activated microglia, we uncovered a large increase of moDC, a threefold decrease of cDC1 and a twofold increase of cDC2. A trend towards this modification of proportions between subsets was already detectable at day 21, i.e., after 7 days of Bev treatment.

Bev decreases cDC1 density, a subset necessary for the development of anti-tumor immunity. These cells excel at cross-presentation [35], are migratory and the main interaction partners of antigen-specific CD8^+^ T cells [36] and have been reported to induce protective immunity in cancers. It is thus possible that their decrease induced by Bev participates to the escape of the tumor treatment by lowering the anti-tumor T cell adaptive response. The maturation of P1 monocytes towards moDC could differ as we observed more LysM-EGFP^+^/CD11c-EYFP^+^ double-labeled cells in treated tumors. moDC, also expressing MHCII, are increased in Bev-treated tumors. Notably, other tumor models with a high moDC content also harbor relatively more MHCII^+^ cells, suggesting that the microenvironment in these tumors favors the differentiation of infiltrating monocytes towards these MHCII^+^ DCs exhibiting immunosuppressive capacities [37]. Notably, the use of moDC to promote protective immunity in patients suffering from infections or cancer have shown moDC limited efficacy, owing to their short life, poor migratory properties, and recirculation to lymph nodes.

Surprisingly, our observations on tissue sections and brain clearing revealed that the EYFP^+^ population migrates in the parenchyma following specific routes such as the corpus callosum. There are two non-exclusive possibilities that would deserve further studies. One is that the population of EYFP fluorescent DCs, never seen in the blood vessels, enters the parenchyma *via* the choroid plexuses [38] and then follows this route to finally accumulate in the lower parts of the tumor (Fig. 5b). Another one is that migratory cDCs use this route to reach the choroid plexus then join the lymph nodes.

It is conceivable that this dynamic adjustment of DC subsets induced by Bev treatment we distinctively visualize in vivo reflects several modifications of physical and chemical communication between innate immune cells and tumor cells, favoring the escape of the tumor to VEGF blockade [39]*.*

## Conclusion

Although the pathogenesis of the tumor and its mutational landscape will arguably differ in our mouse tumor model compared to the in situ tumorigenesis and progression in human tumors, the actual progression of the vascular network and the immune cell recruitment appear highly similar [15, 40]. In the context of tumor therapy, our data shows that VEGF blockade led to an increased recruitment of monocytes and to an adjustment of DC subset profiles, differing in their ability to induce an adaptive immune response and provide important new insights into the spatio-temporal evolution of intra-tumoral innate immune cell densities and directions for further investigations of the functional outcome of their manipulation.

## Additional files

Additional file 1: **Figure S1.** (**A**) Comparison of the mean tumor area at D14 before starting treatment for the two mouse groups (vehicle or Bev). Right panel: example of widefield fluorescent image used to determine the tumor size. Scale bar: 100 μm. (**B**) In vivo 2P imaging showing tumoral cells (red) and vasculature (white) over 250 μm depth (projection max). Blood vessel densities inside and outside tumor volume were calculated using Imaris software (v9.1). A 3D mask was first created in order to define tumor border and, according to blood vessel location (in or out tumor), two distinct masks were then generated to outline the tumoral or peritumoral blood vessels. The vessel density is calculated in number of voxels (present in vessel in or vessel out) over tumoral or peritumoral volume. Scale bar: 200 μm. (**C**) Immunostaining of laminin (white) cells in sagittal sections of tumor bearing brains at D28 treated either with vehicle or Bev. Scale bar: 100 μm. (**D**) Fluorescence immunohistochemistry and confocal microscopy of a vehicle and a Bev-treated tumor at D28, showing neutrophils (LysM-EGFP^+^ Ly6G^+^ cells, white arrows). Scale bar: 50 μm. (PNG 1733 kb)

Additional file 2: **Figure S2.** Impact of Bev-treatment on LysM-EGFP^+^ cells number in blood circulation. Maximal intensity projections of a vehicle (**A**) and a Bev-treated tumor (**B**) at D28, showing the number of LysM-EGFP^+^ cells travelling in blood vessels. Scale bar: 100 μm. (PNG 5327 kb)

Additional file 3: **Figure S3.** Brain slices for fluorescence immunohistochemistry and confocal microscopy. **(A)** Intra-tumoral CD11c-EYFP^+^ cell densities defined in subsets expressing either MHCII^+^ and LysM-EGFP^+^ (left panel) or Iba1^+^ and TMEM119^+^ (right panel) both for vehicle (*n*=2) and Bev-treated mice (n=2). (**B**) CD11c-EYFP^+^/MHCII^+^ cells can be found in choroid plexus (red arrows, zoom inset) as well as single labeled MHCII^+^ cells (blue arrows, left panel). LysM-EGFP^+^ cells are observed in blood vessels (white arrows, right panel) and in choroid plexus (green arrow). Both CD11c-EYFP^+^ and LysM-EGFP^+^ are present in the transition zone between lateral ventricle and choroid plexus among ependymal cells, and can be found in cerebrospinal fluid in ventricle or in brain interstitial space. Scale bars: 50 μm. (**C**) Images of choroid plexus showing CD11c-EYFP^+^/Iba1^+^ (orange arrows), Iba1^+^ cells (magenta) and Iba1^+^/TMEM119^+^ cells in healthy brain parenchyma zone (same slice, right panel). (PNG 2128 kb)

Additional file 4: **Video S1.** Video shows LysM-EGFP^+^ cells trafficking inside vessels (magenta) in untreated mice at day 28. Note that LysM-EGFP^+^ cells are carried away quickly by the blood flow. (MP4 8541 kb)

Additional file 5: **Video S2.** Video shows LysM-EGFP^+^ cells trafficking inside vessels (magenta) in Bev-treated mice at day 28. Note that LysM-EGFP^+^ cells are more numerous with Bev treatment. (MP4 8813 kb)

Additional file 6: **Video S3.** Video shows cell-cell interactions. A LysM-EGFP^+^ cell physically interacts with a CD11c-EYFP^+^ cell. (MP4 2655 kb)

Additional file 7: **Video S4.** Video shows cell-cell interactions. A LysM-EGFP^+^ cell contacts twice a tumor-DsRed cell. (MP4 2650 kb)

Additional file 8: **Table S1.** Antibodies used for multiparametric cytometry experiments. (PNG 118 kb)

## Data Availability

The datasets generated and analyzed during the current study are available from the corresponding author on reasonable request.

## Abbreviations

Bev: Bevacizumab
DC: dendritic cells
FACS: Fluorescence-Activated Cell Sorting
GBM: Glioblastoma
MHC: Mouse Histocompatibility antigen
VEGF: Vascular Endothelial Glial Factor
