# Supplementary figures and images for: Effects of VEGF blockade on the dynamics of the inflammatory landscape in glioblastoma-bearing mice
Source: J Neuroinflammation. 2019 Oct 28;16:191. doi: 10.1186/s12974-019-1563-8 (PMC6816183; doi:10.1186/s12974-019-1563-8)

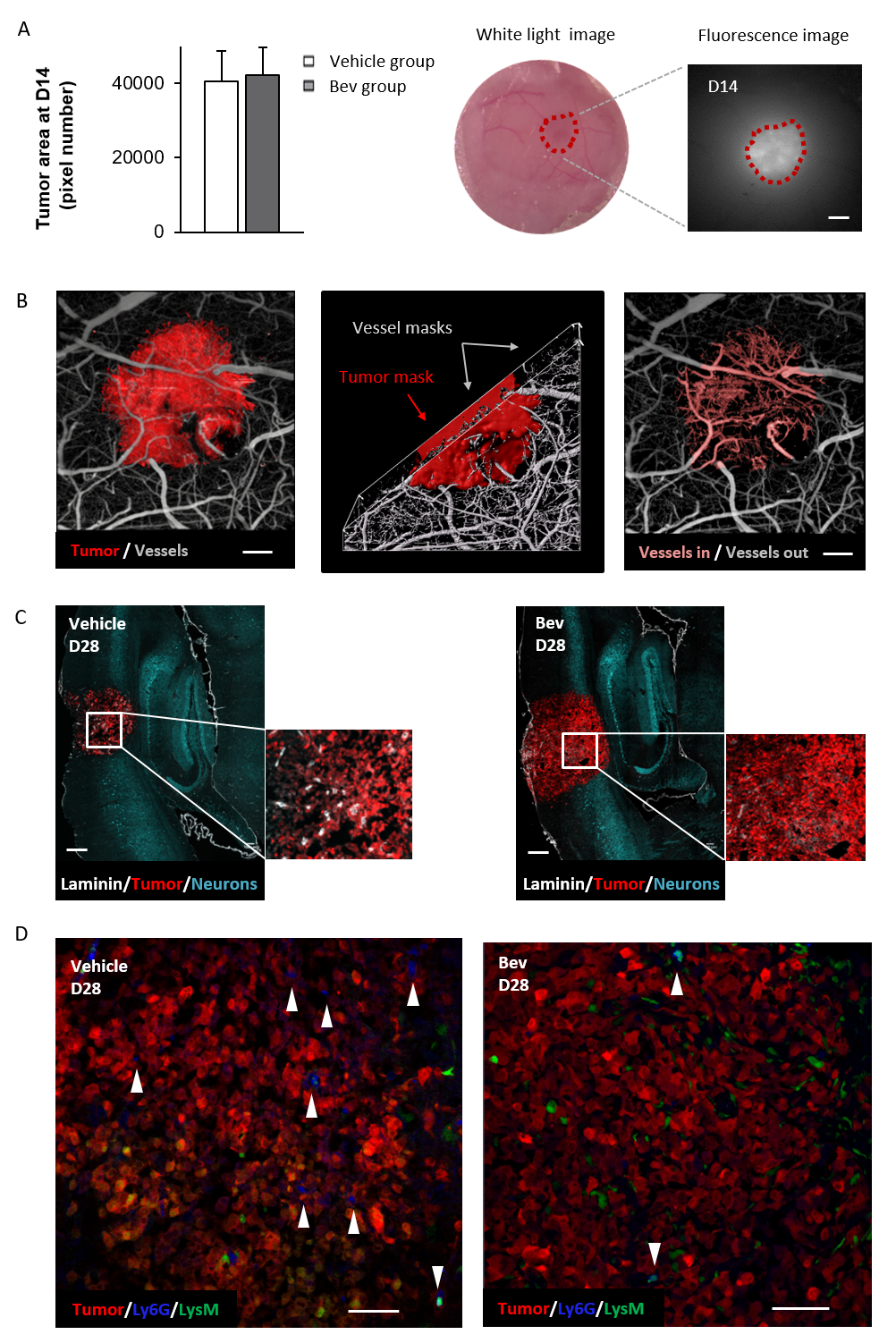

Supplement: Supplementary file 1 — Figure S1. (A) Comparison of the mean tumor area at D14 before starting treatment for the two mouse groups (vehicle or Bev). Right panel: example of widefield fluorescent image used to determine the tumor size. Scale bar: 100 μm. (B) In vivo 2P imaging showing tumoral cells (red) and vasculature (white) over 250 μm depth (projection max). Blood vessel densities inside and outside tumor volume were calculated using Imaris software (v9.1). A 3D mask was first created in order to define tumor border and, according to blood vessel location (in or out tumor), two distinct masks were then generated to outline the tumoral or peritumoral blood vessels. The vessel density is calculated in number of voxels (present in vessel in or vessel out) over tumoral or peritumoral volume. Scale bar: 200 μm. (C) Immunostaining of laminin (white) cells in sagittal sections of tumor bearing brains at D28 treated either with vehicle or Bev. Scale bar: 100 μm. (D) Fluorescence immunohistochemistry and confocal microscopy of a vehicle and a Bev-treated tumor at D28, showing neutrophils (LysM-EGFP+ Ly6G+ cells, white arrows). Scale bar: 50 μm. (PNG 1733 kb) [file 12974_2019_1563_MOESM1_ESM.png]

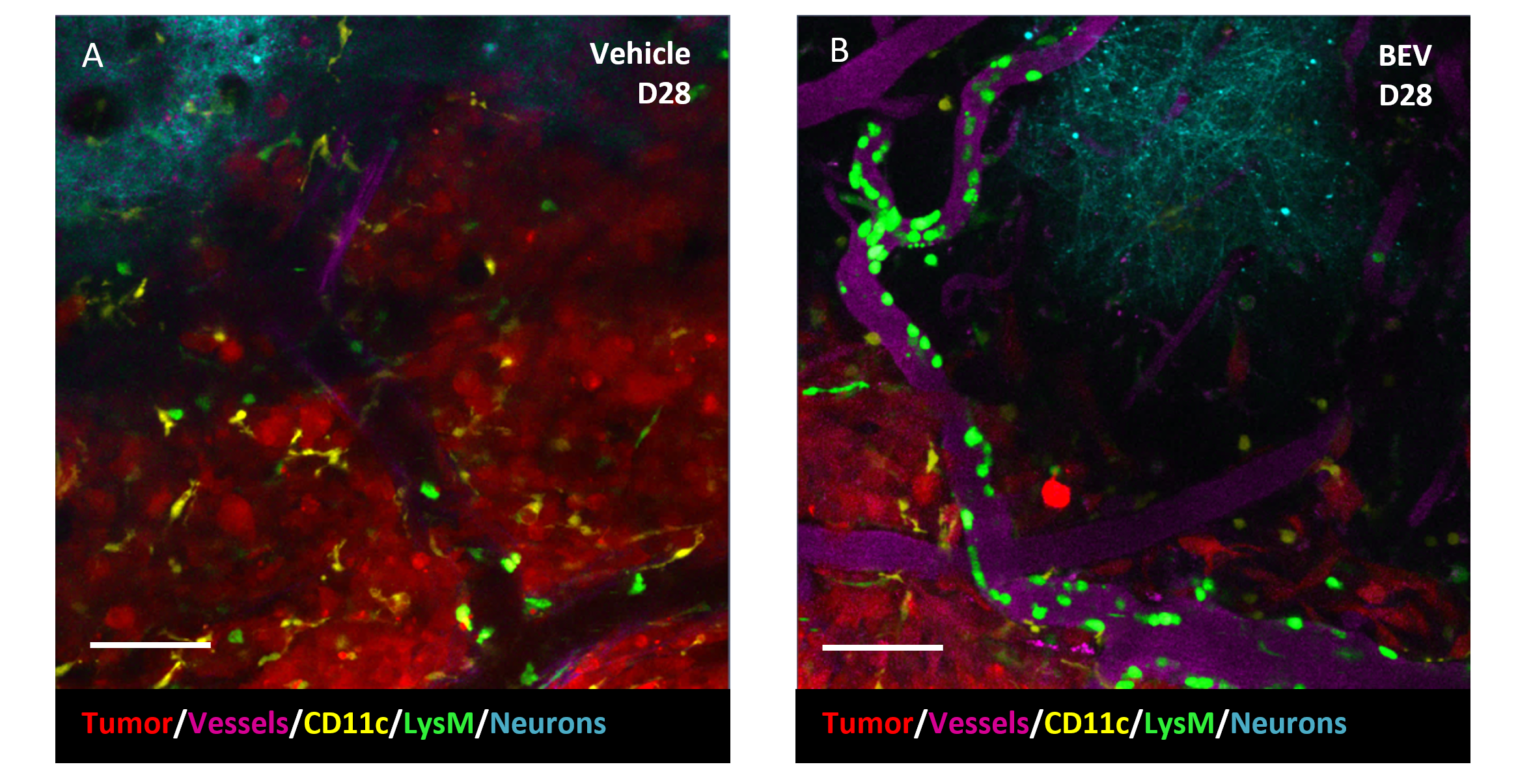

Supplement: Supplementary file 2 — Figure S2. Impact of Bev-treatment on LysM-EGFP+ cells number in blood circulation. Maximal intensity projections of a vehicle (A) and a Bev-treated tumor (B) at D28, showing the number of LysM-EGFP+ cells travelling in blood vessels. Scale bar: 100 μm. (PNG 5327 kb) [file 12974_2019_1563_MOESM2_ESM.png]

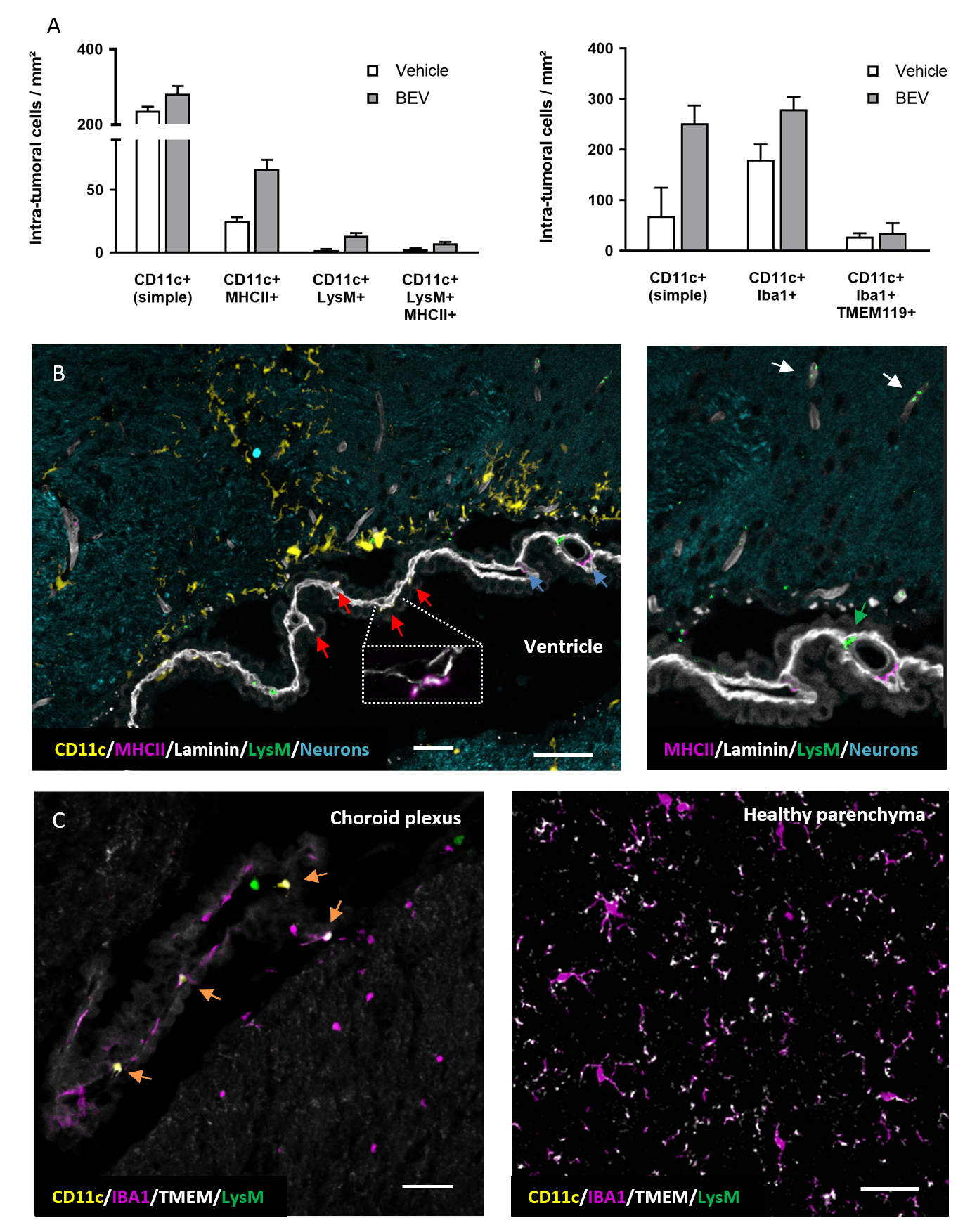

Supplement: Supplementary file 3 — Figure S3. Brain slices for fluorescence immunohistochemistry and confocal microscopy. (A) Intra-tumoral CD11c-EYFP+ cell densities defined in subsets expressing either MHCII+ and LysM-EGFP+ (left panel) or Iba1+ and TMEM119+ (right panel) both for vehicle (n=2) and Bev-treated mice (n=2). (B) CD11c-EYFP+/MHCII+ cells can be found in choroid plexus (red arrows, zoom inset) as well as single labeled MHCII+ cells (blue arrows, left panel). LysM-EGFP+ cells are observed in blood vessels (white arrows, right panel) and in choroid plexus (green arrow). Both CD11c-EYFP+ and LysM-EGFP+ are present in the transition zone between lateral ventricle and choroid plexus among ependymal cells, and can be found in cerebrospinal fluid in ventricle or in brain interstitial space. Scale bars: 50 μm. (C) Images of choroid plexus showing CD11c-EYFP+/Iba1+ (orange arrows), Iba1+ cells (magenta) and Iba1+/TMEM119+ cells in healthy brain parenchyma zone (same slice, right panel). (PNG 2128 kb) [file 12974_2019_1563_MOESM3_ESM.png]

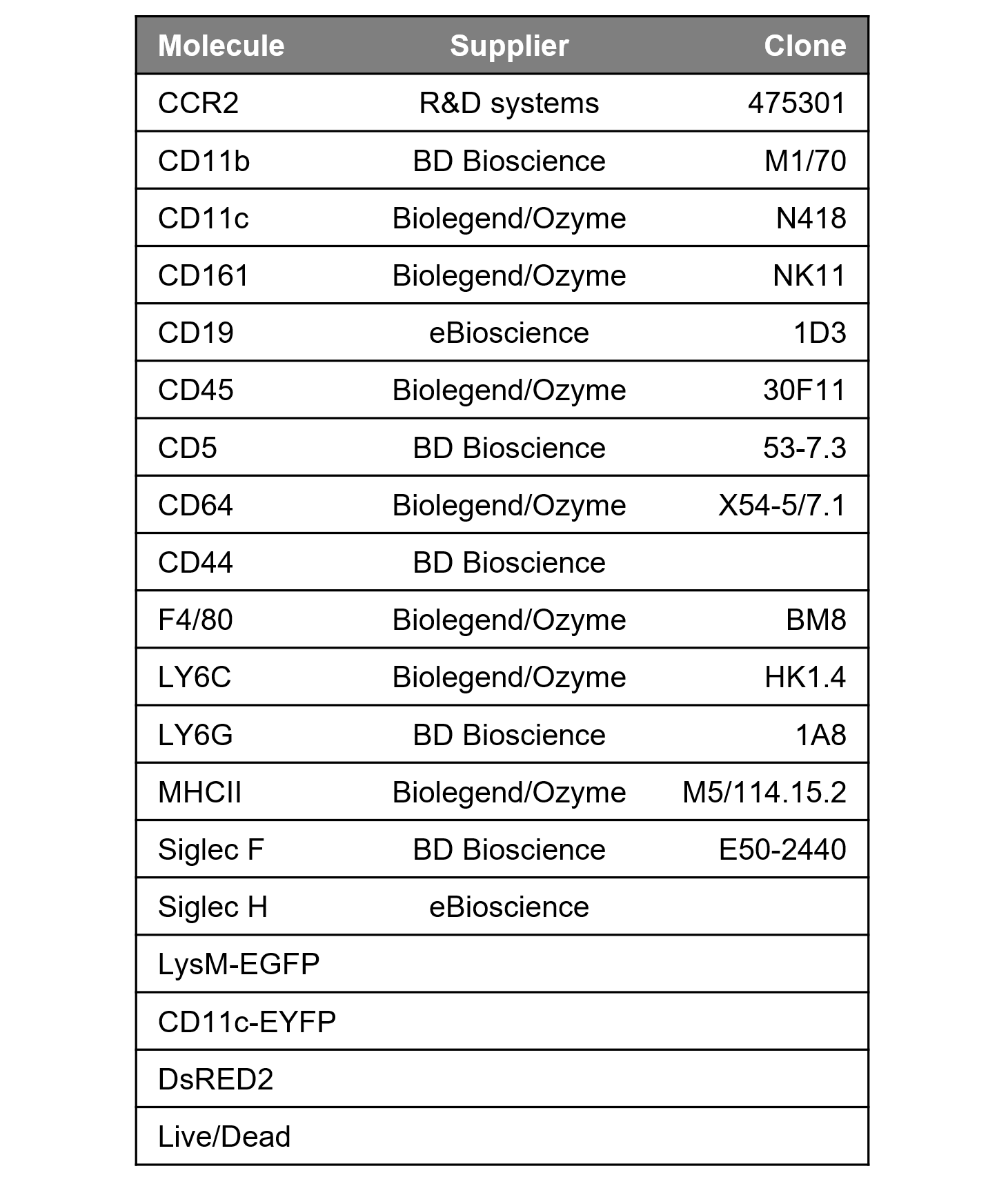

Supplement: Supplementary file 8 — Table S1. Antibodies used for multiparametric cytometry experiments. (PNG 118 kb) [file 12974_2019_1563_MOESM8_ESM.png]
